# Supplementary material for: The recovery of parabolic avalanches in spatially subsampled neuronal networks at criticality
Source: Sci Rep. 2024 Aug 20;14:19329. doi: 10.1038/s41598-024-70014-4 (PMC11335857; doi:10.1038/s41598-024-70014-4)
Supplement: Supplementary file 1 — Supplementary Information. [file 41598_2024_70014_MOESM1_ESM.docx]

**The recovery of parabolic avalanches in spatially subsampled neuronal networks at criticality**

# Supplementary Information

Keshav Srinivasan, Tiago L. Ribeiro, Patrick Kells, and Dietmar Plenz^†^
Section on Critical Brain Dynamics, National Institute of Mental Health, Bethesda, MD 20892, USA

^†^ Correspondence

**
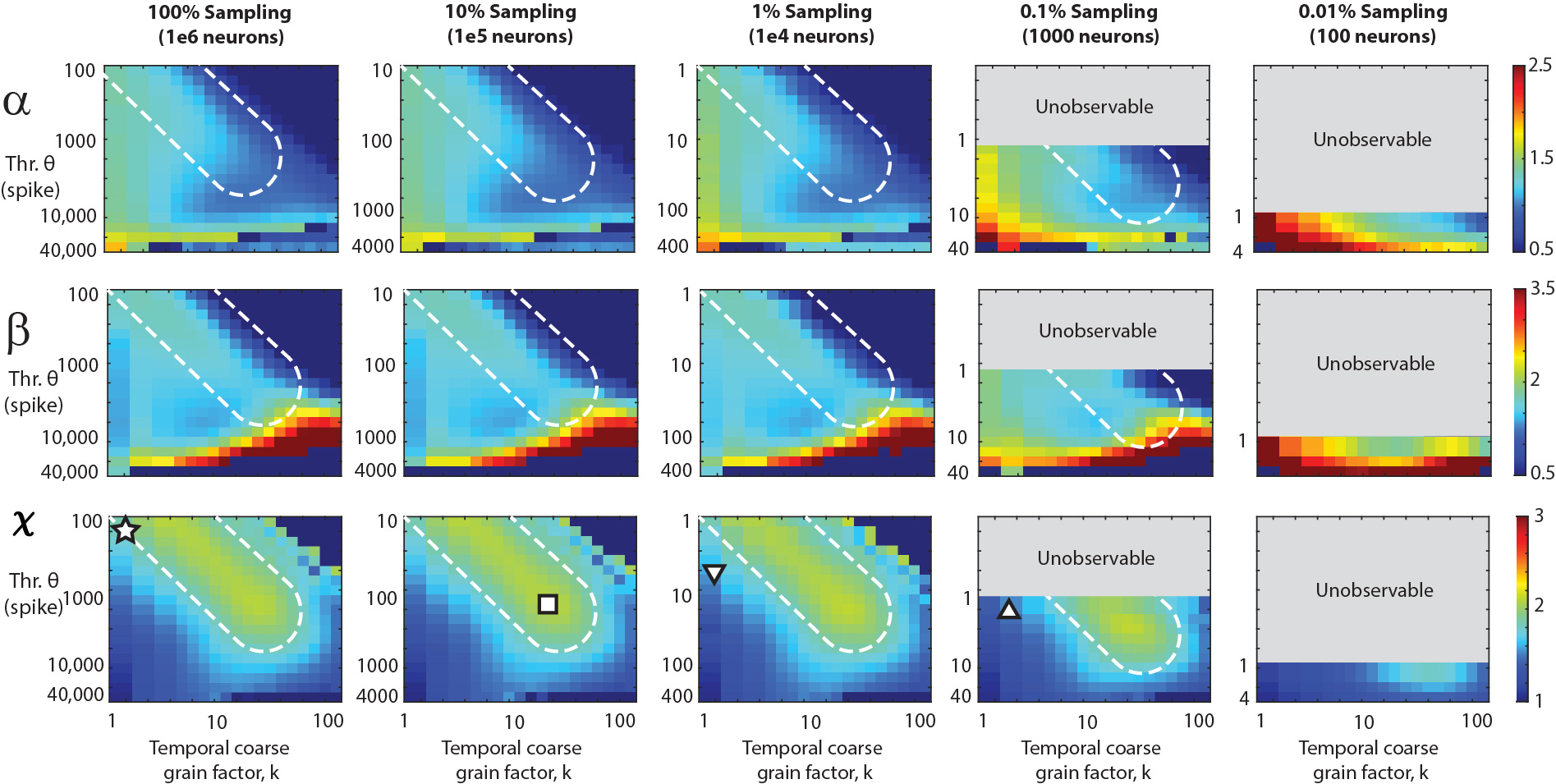
**

**Suppl. Fig. S1. Consolidated view of the exponents α, β and 𝜒 as a function of threshold and temporal coarse-graining for different sampling fractions.**

Consolidated view of α, β and *χ* as a function of *θ* and *k* for different values of *f*. For *f* = 100%, *χ* ≅ 2 for low *θ* and *k* (*star*) but as we make the data sparser by increasing the threshold (or reducing the sampling), we need a higher coarse graining factor, *k*, to compensate and rescue *χ* back to 2 (*square*). Bottom row is replotted for *χ* from Figure 4 for ease of comparison with the corresponding size slopes, *α*, and duration slopes, *β*. *White dotted region*: visual guide for *χ* close to 2 for increasing *θ* and *k*. The grey parts of the plots for f = 0.1% and 0.01% respectively are unobservable parameter regions since they would require fractional thresholds, below the 1 spike minimum resolution of the model (*cf.* Figure 4).
